# Disease resistance and infectivity of virus susceptible and resistant common carp strains

**DOI:** 10.1038/s41598-024-55133-2

**Published:** 2024-02-26

**Authors:** Batya Dorfman, Evgeniya Marcos-Hadad, Roni Tadmor-Levi, Lior David

**Affiliations:** Department of Animal Sciences, RH Smith Faculty of Agriculture, Food and Environment, The Hebrew University of Jerusalem, Rehovot, Israel

**Keywords:** Viral infection, Animal breeding

## Abstract

Infectious diseases challenge health and welfare of humans and animals. Unlike for humans, breeding of genetically resistant animals is a sustainable solution, also providing unique research opportunities. Chances to survive a disease are improved by disease resistance, but depend also on chances to get infected and infect others. Considerable knowledge exists on chances of susceptible and resistant animals to survive a disease, yet, almost none on their infectivity and if and how resistance and infectivity correlate. Common carp (*Cyprinus carpio*) is widely produced in aquaculture, suffering significantly from a disease caused by cyprinid herpes virus type 3 (CyHV-3). Here, the infectivity of disease-resistant and susceptible fish types was tested by playing roles of shedders (infecting) and cohabitants (infected) in all four type-role combinations. Resistant shedders restricted spleen viral load and survived more than susceptible ones. However, mortality of susceptible cohabitants infected by resistant shedders was lower than that of resistant cohabitants infected by susceptible shedders. Virus levels in water were lower in tanks with resistant shedders leading to lower spleen viral loads in cohabitants. Thus, we empirically demonstrated that disease resistant fish survive better and infect less, with implications to epidemiology in general and to the benefit of aquaculture production.

## Introduction

The prevention and treatment of infectious diseases is an ever-evolving challenge. Types of pathogens might change among hosts and over time, but what commonly govern the dynamics of a disease outbreak are host–pathogen interactions^1–3^. Controlling infectious disease outbreaks is of utmost significance for the health of humans, but infectious pathogens present significant challenges to other animals as well, particularly to sustainable farming of animals^4–9^. Number of farmed animals have been increasing to meet food demands and their stocking densities are high to improve efficiency. Aquaculture suffers most, as animal numbers and stocking densities are exceptionally high, pathogens spread efficiently in water, and means to prevent and control disease outbreaks under aquaculture conditions are limited^10,11^. However, with respect to disease control, unlike for humans, developing disease-resistant animals is a highly desirable, sustainable and cost-effective solution to mitigate the devastating consequences of infectious disease outbreaks^12–14^. Notably, even at random, some susceptible individuals might stand a chance to survive even a severe disease, implying that survivors are not necessarily resistant. Chances of disease resistant animals to survive are higher because of their genetics, and once trait variation exists, then breeding of resistant strains is feasible^15–17^.

Understanding disease resistance mechanisms has not only been promoting sustainable farming solutions. Having susceptible and resistant strains in hand also allows their comparison, forming an indispensable research system to enhance our knowledge on how different host–pathogen interactions lead to different disease outcomes^3,18–22^. Disease resistance mechanisms are better studied upon the first host–pathogen encounter, since upon a repeated exposure of survivors, their immune memory can play a major role obscuring the resistance mechanism^14^. The most common way used to evaluate disease resistance is measuring survival/mortality from a disease. This estimator is rough, since this binary phenotype could have a complex genetic basis^23–26^. In addition, the commonly used term “disease resistance” is ought to be more specifically defined depending on the underlying mechanism^27–29^. For instance, disease resistance is not necessarily the same as disease tolerance or infection resistance, since different mechanisms might play a role in restricting the pathogen replication in host tissues, tolerating host tissue damages or limiting pathogen entry into a host, respectively^30–34^. Importantly, the mechanisms leading to surviving a disease are not necessarily mutually exclusive or controlled by a single gene, often making survival chances a quantitative rather than a binary trait^15,31,35^.

The dynamics of disease outbreaks is affected not only by the host’s resistance but also by its tendency to infect others (infectivity)^13,31,36–38^. A reasonable and common hypothesis is that disease resistant individuals are less likely to become sick and thus, will be less infective than susceptible individuals. On the other hand, it was hypothesized that certain infected, sick or asymptomatic, individuals can play a role of super-spreaders^38–42^. Although an important aspect in disease epidemiology, these hypotheses on infectivity were tested mostly by epidemiological models^43–45^. Adjusting models to include indirect genetic effects allowed the incorporation of heritable traits belonging to one individual and their effects on the traits of another^36,46–49^. However, although there are hypotheses on and modeling of infectivity, so far, only a few studies evaluated empirically the relationship between disease resistance and infectivity^46,49^.

The common carp, *Cyprinus carpio* Linnaeus, 1758, is among the most highly produced freshwater fish species globally^50^. Both food carp and ornamental Koi strains are highly susceptible to an illness caused by the cyprinid herpes virus type 3 (CyHV-3, also known as Koi Herpesvirus or KHV). The disease was first recorded in the 1990s and has since spread to common carp populations wherever cultured^51–56^. Belonging to the Alloherpesviridae family^57^, this dsDNA virus affects epithelial cells, causing tissue necrosis and hemorrhages of the gills and liver, behavioral changes, and mass mortality. Susceptible fish exposed to CyHV-3 under permissive temperatures (18–26 °C) die within 6–21 days post-infection, and the losses are mounting to 60–100% of all fish^58–61^.

Resistance to CyHV-3 was identified in feral strains^62–66^. Our group has been breeding for CyHV-3 disease resistance by trait introgression from a resistant feral strain into two susceptible food strains, followed by backcrosses and family selection of fish from resistant families^65^. Previous studies indicated a considerable additive genetic component for CyHV-3 resistance^65,67,68^. Several genomic regions (QTLs) affecting fish survival were identified, indicating CyHV-3 is a polygenic trait, and several immune system genes were identified inside these QTLs, suggesting the involvement of immunity in resistance^52,68–71^.

In this study, by using an infection-by-cohabitation model, susceptible and resistant fish types played roles as shedders (infecting) or cohabitants (infected) in all four combinations of type and role. Mortalities, tissue viral loads and tank water viral loads were recorded to follow the path of infection and disease and study if and how disease resistance variation relates to infectivity differences.

## Results

### Mortality patterns

**Figure 1:**
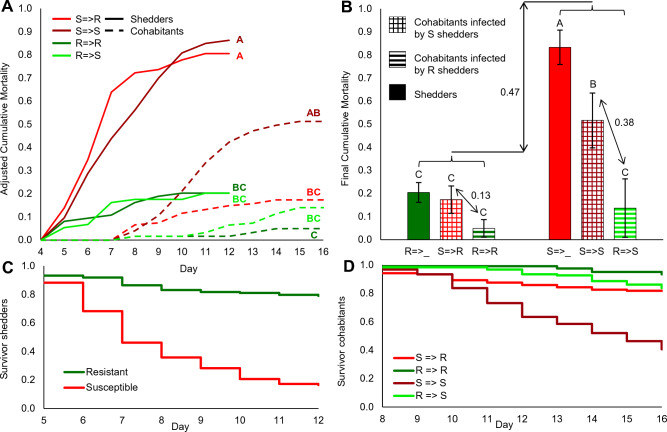
Cumulative mortality of experimental groups. (**A**) Mean cumulative mortality by days (adjusted to start day) for shedders (full lines) and cohabitants (dashed lines). S for susceptible (red shades), R for resistant (green shades) and arrow for the direction of infection (shedder => cohabitant). (**B**) Final mean cumulative mortalities and standard errors for resistant (left bars) and susceptible (right bars) categories. Within each category, the left bar (full color) is for shedders and the two right bars (meshed and striped) are for cohabitants infected by different shedders (susceptible in red and resistant in green). Different letters denote statistically different means. Arrows denote differences between means. Survival analysis for (**C**) shedders, right censored to day 12 and (**D**) cohabitants, right censored to day 16. Note that survival analyses results are similar to mortality analyses presented in (**A**).

Cumulative mortality curves of the four treatments were produced separately for shedders and cohabitants, by averaging counts of the four replicates (Supp. Fig. 1) and after adjustments for untimed mortalities and mortality start day (Fig. 1A). Shedders, which were all equally infected with virus by IP injection, suffered mortalities as of day 5 post-infection and progressively accumulated more as expected from their type. Final cumulative mortality was significantly higher for susceptible compared to resistant shedders (means of 0.83 and 0.20, *t* test, *P* < 0.0001), validating the disease model and the difference between fish types. For cohabitants, mortalities started 3 days later, at day 8. However, cohabitant mortalities accumulated depending on the combination of shedder and cohabitant types (Fig. 1A). Final cumulative mortality was highest for susceptible cohabitants infected by susceptible shedders (mean = 0.52) and lowest for resistant cohabitants infected by resistant shedders (mean = 0.05), as might be expected. However, resistant cohabitants infected by susceptible shedders (mean = 0.17) suffered slightly higher mortalities than susceptible cohabitants infected by resistant shedders (mean = 0.14). Statistically, S => S had significantly higher cumulative mortality than R => R (Tukey–Kramer HSD, *P* = 0.019), whereas the other two treatments were not different from each other or from susceptible shedders (Fig. 1A). Further, survival analyses on the same data confirmed the differences for both the shedders and cohabitants (Log-Rank, *P* < 0.0001 for both, Fig. 1C and D). Thus, chances of host mortality relied on not only its own type but also the type it was infected by.

Furthermore, for each fish type (susceptible or resistant), three final cumulative mortality means were calculated: one of shedders and two of cohabitants by what shedder type they were infected by (Fig. 1B). The mean mortality of all susceptible categories was higher by 0.47 than that of all resistant categories combined. In more details, susceptible shedders had cumulative mortality (0.83) significantly higher than susceptible cohabitants infected by susceptible shedders (0.52), and both had significantly higher mortalities than the other four categories (0.20–0.05), including from susceptible infected by resistant (Tukey–Kramer HSD, *P* < 0.0001). Thus, mortality of cohabitants was consistently lower when infected by resistant shedders. Switching shedders from susceptible to resistant reduced mortalities by 0.13 and 0.38, for resistant and susceptible cohabitants, respectively. From the perspective of final mortality, these analyses indicated that infection by cohabitation is less severe compared to that by IP injection and more effective by susceptible fish.

### Relative viral load in spleen

**Figure 2:**
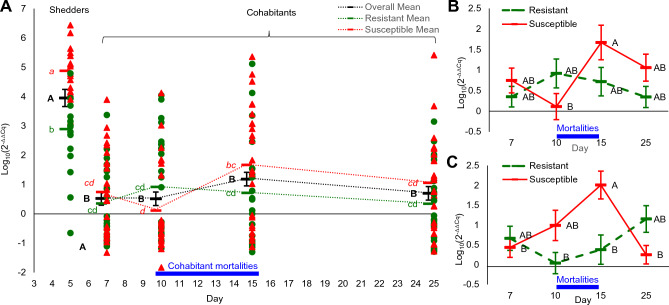
Relative viral loads (Log_10_(2^–ΔΔCq^)) in spleen by day and fish type. (**A**) Individual values of resistant in green dots and of susceptible in red triangles by day post infection. Overall means (black bars) and means of susceptible (red bars) and resistant fish (green bars). Day 5 in shedders and days 7–25 in cohabitants. Means of different days of the same cohabitant type are connected by dotted lines. Different letters denote statistically different means (*P* < 0.05). Overall means were tested separately from group means. (**B**) Relative viral loads in spleen of cohabitants by cohabitant type and days. (**C**) Relative viral loads in spleen of cohabitants by shedder type and days. Presented are means and standard errors. Different letters denote statistical significance (*P* < 0.05). Blue line on the X-axis denotes days of cohabitant mortalities. Note that shedder type (panel (**C**)) affects viral loads in cohabitant spleen more than the own cohabitant type (panel (**B**)).

Viral loads were analyzed in spleen of live fish only, including from sampling days with concurrent mortalities, and thus, it is likely that viral levels measured were under-estimated, since fish that died in sampling days and earlier are likely to have had even higher viral loads. Relative viral loads in shedders were measured at day 5 post injection, allowing time for tissue infection, but before significant infection by cohabitation with peers occurred. Relative viral loads in cohabitants were measured later at days 7, 10, 15 and 25 to follow the cohabitation effect. Despite being injected with equal amounts of virus, by day 5, viral load in susceptible shedders (mean = 4.88) was 100 times higher than in resistant ones (mean = 2.89; *t* test, log_10_ scale, *P* < 0.0001; Fig. 2A). Mean viral load in shedders at day 5 (3.95, resistant and susceptible combined) was significantly higher than all loads measured in cohabitants, both on average and when resistant and susceptible fish were separately compared. In particular, load in shedders at day 5 was 2600 and 570 times higher than in cohabitants at days 10, (mortality start, mean = 0.52) and 15 (mortality peak, mean = 1.19), respectively (Tukey Kramer HSD test, *P* < 0.0001; Fig. 2A). Further, viral loads of injected shedders at day 5, among both susceptible and resistant fish, were less variable than those among cohabitants (Fig. 2A).

To understand better the dynamics of spleen viral loads, relative levels of only cohabitants were analyzed further by a full factorial model, considering the effects of day, shedder type and cohabitant type. A significant interaction was found between day and cohabitant type (*P* = 0.025). Viral load in susceptible cohabitants significantly increased towards day 15 and decreased back towards day 25 (Tukey–Kramer HSD, *P* = 0.0194), whereas in resistant cohabitants viral loads did not vary significantly over days (Fig. 2B). Further, the effect of shedder type on viral loads in cohabitants was analyzed, yet again, the interaction with day was significant (*P* = 0.00024, Fig. 2C). Cohabitants infected by susceptible shedders had a gradual and significant increase in viral loads from day 7 to day 15 (Tukey–Kramer, *P* = 0.0127) and a significant decrease back to day 25 (Tukey–Kramer, *P* = 0.003), whereas cohabitants infected by resistant shedders had lower and steadier loads overall, and significantly lower than those infected by susceptible shedders at day 15 (Tukey–Kramer HSD, *P* = 0.0083).

Thus, infection by IP injection led to much higher and more uniform viral loads in host tissues compared to infection by cohabitation. In addition, resistant fish restricted viral replication much more effectively than susceptible ones. Finally, spleen viral loads in live fish infected by cohabitation increased alongside mortalities and were affected significantly not only by the type of host fish, but also by the type of infecting fish.

### Relative DNA loads in tank water

**Figure 3:**
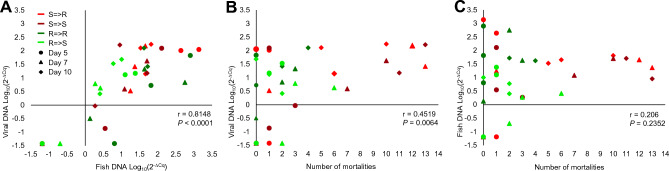
Correlations between mortality and relative DNA loads (Log_10_(2^–ΔCq^)) in tank water. (**A**) Between relative viral DNA loads (Y-axis) and fish DNA loads (X-axis) in the water. (**B**) Between relative viral DNA load (Y-axis) and total number of mortalities around the water sampling days. (**C**) Between relative fish DNA load (Y-axis) and total number of mortalities around the water sampling days. Shown are correlation coefficients and their *P* values comparing to 0. Each point is replicate measurement of treatment (by colors) and day (by shape).

Implied by the cohabitation infection model, shedders were likely infecting cohabitants primarily by releasing virus particles into the environment. Therefore, viral loads and fish DNA loads, were measured in water samples taken from tanks on days 5, 7 and 10, the period at which infection by cohabitation was expected and overlapping the early measurements of viral loads in spleen of hosts. Firstly, to understand what affects viral and fish DNA loads in water, three parameters were correlated (Fig. 3). A strong and positive correlation was found between viral and fish DNA loads in water (r = 0.8148, *P* < 0.0001; Fig. 3A). Next, a medium and positive correlation was found between viral load and number of mortalities around the water sampling day (r = 0.4519, *P* = 0.0064; Fig. 3B). Lastly, no correlation was found between fish DNA load and number of mortalities around the water sampling day (r = 0.206, *P* = 0.2352; Fig. 3C). These correlations suggested that release of DNAs into the water relates to mortalities, more for viral than for fish DNA. It is also possible that release of both DNA types relates to tissue damage leading to mortalities.

**Figure 4:**
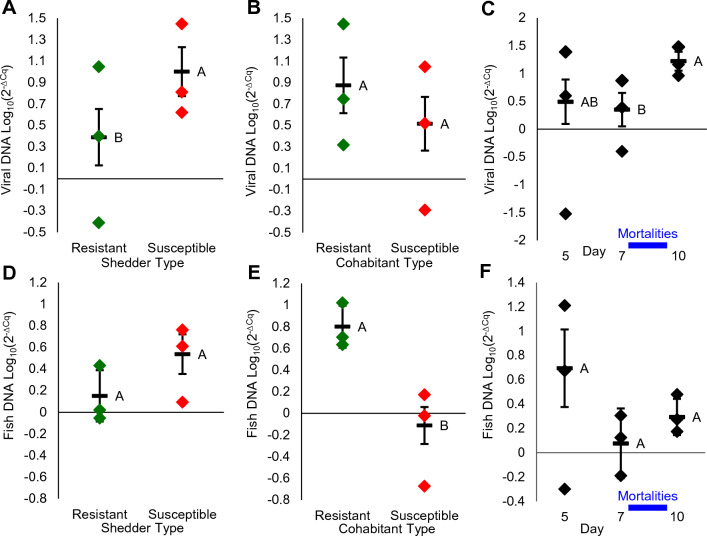
Effects of shedder type, cohabitant type and day on relative DNA loads (Log_10_(2^–ΔCq^)) in tank water. (**A**–**C**) Relative viral DNA load (Y-axes) by shedder type (**A**), cohabitant type (**B**) (resistant in green and susceptible in red) and overall fish by day (**C**). (**D**–**F**) Relative fish DNA load (Y-axes) by shedder type (**D**), cohabitant type (**E**) and overall fish by day (**F**). Shown are three replicates, overall means and standard errors. Different letters denote statistical significance (*P* < 0.05). In blue line are days of main shedder mortalities. Note that susceptible shedders release more virus to the water (panel (**A**)), viral load in water increase in parallel to mortalities (panel (**C**)) and fish DNA load was higher in tanks of resistant cohabitants.

To understand better what affects DNA levels in the water, a full factorial model was applied for viral DNA separately from fish DNA loads in tank water. The model interactions for both fish and virus DNA loads were insignificant and thus, main factors were analyzed individually. Interestingly, higher viral loads were found in tanks of susceptible shedders compared to tanks of resistant ones (*t* test, *P* = 0.0445; Fig. 4A), but no significant differences between tanks of susceptible and resistant cohabitants (*t* test, *P* = 0.3276; Fig. 4B). Further viral loads in water significantly increased between day 7 and 10, in line with the end of shedders’ and start of cohabitants’ mortalities (Student’s *t* test, *P* = 0.0447; Fig. 4C). With respect to fish DNA loads in water, a trend was observed for higher levels in tanks of susceptible shedders (*t* test, *P* = 0.1039; Fig. 4D), but a significantly higher level was found in tanks of resistant cohabitants compared to susceptible ones (*t* test, *P* = 0.0014; Fig. 4E). Fish DNA loads were similar among sampling days (Student’s *t* test, *P* = 0.1046; Fig. 4F). Thus, although correlated, viral DNA levels in water were affected more by shedder type, whereas fish DNA levels more by cohabitant type, indicating that susceptible fish shed more virus, especially around days with mortalities.

## Discussion

The aim of this study was to experimentally analyze if and how variation in disease resistance relates to infectivity differences under the infection-by-cohabitation model. But first, since the adjective resistant is generally used, two distinctions are in place in the context of this study. Resistant are not individuals merely surviving a disease, simply because survivors are found, although to a lesser extent, also in susceptible groups. Resistant are also not individuals immunized by prior infection or vaccination, since immunized individuals might be protected by a different mechanism than that protecting disease resistant fish upon their first exposure. Here, disease resistant fish have a genetic heritable trait significantly improving the chances of naïve individuals to survive the disease upon their first encounter with the pathogen^65,71,72^.

We experimented using both IP injection of shedders, ensuring their equal infection, and infection-by-cohabitation for cohabitants, mimicking natural infection conditions. Hence, our setup allows addressing differences related to infection and infectivity. Evidently, the infection method matters, since both tissue viral loads (Fig. 2) and mortalities (Fig. 1) were lower in cohabitants (infected by cohabitation) than in shedders (infected by IP injection). Our previous findings already demonstrated that resistant fish can be infected by cohabitation, but they restrict the tissue viral load better than susceptible fish^65^. In this study, shedders were all equally IP injected and again resistant fish constrained viral replication two orders of magnitude better than susceptible ones (Fig. 2). Thus, infection by injection confirmed the resistance mechanism, which is consistent with the definition of disease resistance being the improved capacity of the host to restrict the viral load and its consequent tissue damages^14,28,32^. Notwithstanding the disease resistance, since both mortalities and spleen viral loads were generally lower in cohabitants compared to shedders (Figs. 1, 2), we cannot rule out that resistant fish are also somewhat infection resistant, although this effect could also be due to the specific conditions of water exchange rate and fish density, enabling some cohabitants to avoid initial infection.

Mortalities in cohabitants followed those of shedders by about 4–6 days (Fig. 1A), again validating the infection-by-cohabitation model, but more importantly, allowing to address questions related to infectivity. Notably, several results suggested that cohabitation, and therefore, infection under natural conditions, rely on the proportion of infecting-to-infected hosts^44,45^. We applied a relatively high ratio of 1:2 between shedders and cohabitants, yet, despite this high ratio and the significant viral propagation in shedders (Fig. 2), the efficiency of infection by cohabitation was incomplete, as supported by three results. First, viral loads in spleen of cohabitants at day 10 were much lower compared to that in shedders at day 5 (Fig. 2). Secondly, cumulative mortality of cohabitants was lower than that of shedders of the same type (Fig. 1). Lastly, records of late mortalities (Supp. Fig. 1) were mainly of cohabitants that, based on their late timing, were probably infected by other cohabitants rather than originally by shedders. Therefore, in aquaculture, and more so under natural conditions, infection will progressively take hold leading to more prolonged outbreaks and to continuous mortalities compared to our controlled and rigorous experimental model.

Furthermore, mortalities of cohabitants revealed an interesting pattern, reflecting the combined effect of shedder and cohabitant types. Ranking cumulative mortality by day 16, placed cohabitants (susceptible and resistant) infected by susceptible shedders above those infected by resistant shedders (Fig. 1). Susceptible cohabitants suffered significant mortalities when infected by susceptible shedders, but significantly less when infected by resistant shedders. In fact, compared to infection by susceptible shedders, infection by resistant shedders reduced cumulative mortality of susceptible and resistant fish by 0.38 and 0.13, respectively (Fig. 1B). These effects estimate infectivity differences, in comparison to a difference in cumulative mortality of 0.6 between susceptible and resistant shedders, estimating the effect of disease resistance alone. Thus, disease-susceptible fish not only had higher mortality rates but also were more infectious than resistant ones.

Higher infectivity was reflected also in viral loads. In shedders at day 5, viral load was 100 times higher in susceptible vs. resistant fish (Fig. 2). This led to 9 and 44 times higher viral loads by days 10 and 15, respectively, in spleen of cohabitants (susceptible and resistant combined) infected by susceptible shedders compared to cohabitants infected by resistant shedders. Consistently, viral loads in water increased till day 10, in correlation with increased number of mortalities, which were higher for susceptible shedders. On average, viral loads in water were four times higher in tanks with susceptible shedders, who suffered more tissue damages and mortalities compared to resistant ones (Fig. 4A). Fish DNA loads were higher in water of tanks with resistant cohabitants, likely because of less mortalities and higher biomass (Fig. 4D). Taken together, in the order actually occurring, genetically susceptible fish are more prone to infection, restrict less the viral replication in their tissues once infected, consequently shed more virus into the water and hence are more infective. As a result, an individual susceptible fish has lower chances to survive an outbreak and as a population, susceptible fish will suffer higher mortality rates and will spread the disease faster and to a higher extent. A resistant population will also suffer higher mortalities when infected by susceptible shedders. This chain of events, negatively relating disease resistance to infectivity, makes sense in the light of the disease resistance mechanism. We already showed that CyHV-3 resistance is a polygenic trait relying on an improved function of the host immune system^71,72^, which restrains the pathogen replication in the host, as was also found in other diseases and hosts^14,73–77^. Here it was shown for the first time that this disease resistance mechanism also leads to reduced infectivity.

Having demonstrated that susceptible fish shed the virus more effectively but are more likely to die from the disease, one might wonder if susceptible fish are actually super-spreaders^39,40,78,79^. Total spreading of the disease depends on amount shed per time unit and overall duration of shedding. Although several studies referred to the possibility of CyHV-3 latency^80–83^, other studies demonstrated the long immune protection that recovered fish hold^84–88^. Our results showed that by day 25, when fish had recovered, spleen viral loads had significantly decreased, hence probably also their shedding. Upon re-exposure, either by re-infection or by activation of a latent virus, survivors, which are immunized, will restrict the virus load, hence the shedding. Thus, our results suggest that the duration of significant shedding is mainly during an outbreak. Thus, based on the results of this study, whom qualifying better to the definition of super-spreaders are susceptible fish during the disease, because of their short but heavier shedding.

Under the premise that resistant fish here are mainly disease-resistant (and likely also slightly infection-resistant), our experiment provides prime empirical evidence that they are also less infectious than susceptible fish. As striking as this result is, it is noteworthy to remember that, in this study (Fig. 2) as in many other epidemiological studies, variation exists between individuals^38^. Some disease-resistant fish might fail to restrict viral replication, become more infective and end up dead, while some susceptible fish may not reach deadly infection levels, stay less infective, recover and become protected. Thus, here, both disease resistance and infectivity are quantitative measurements affecting chances of an individual to survive. In addition, since this disease resistance as a survival mechanism, also reduces infectivity, it bares implications also on survival rates and disease spread on the population level^84,89,90^.

In conclusion, we clearly demonstrated experimentally that resistance to CyHV-3 leads to lower infectivity as a result of a host mechanism that restricts viral replication and hence pathogen shedding. This has significant implications to both aquaculture production and health of populations in natural water bodies. This study amplifies the significance of breeding disease resistant animals as a solution to sustainable food production and animal welfare. In the perspective of disease epidemiology, disease resistant animals are beneficial two folds, since they are less likely to die and are less infective of others. These virtues predict not only that genetically resistant fish will prevail following disease outbreaks, but will also contribute to reducing further virus propagation and disease spread.

## Methods

### Ethics statement

All experiments were carried out in accordance with procedures reviewed and approved in permit #AG-19-15769-5 from the committee for ethics in experiments involving animals of the Hebrew University of Jerusalem. Tissue sampling from live fish was done following euthanasia by immersion in water with high concentration of 2-PhenoxyEthanol. At the end of the challenge, fish that survived were euthanized in the same way. All authors complied with the ARRIVE guidelines for animal experiments.

### Fish experiments

Fish families of food-type carp, produced as part of the CyHV-3 resistance breeding scheme were disease challenged using our infection-by-cohabitation model^65^. Each family was challenged in 3–4 replicates (n = 50 fish per replicate) and its mean cumulative % survival was calculated. From these initial experiments, fish from four resistant families (final cumulative % survival range of 0.61–0.75) were mixed and used as the resistant fish for this experiment. As susceptible fish, groups of Koi fish, which is the ornamental variant of the same species (*C. carpio*), were CyHV-3 challenged together with the food-type fish. Koi groups showed cumulative % survival range of 0.07–0.38, and also fish from these groups were mixed. Thus, food-type fish from resistant families and Koi fish from susceptible groups, which were not yet challenged (naïve), were randomly chosen for this study to serve as resistant and susceptible types, respectively. All fish were about 6-months-old and around 20 g in weight.

In this study, CyHV-3 challenges were carried out in 100 L tanks with a steady air supply, temperature of 22–24 °C and fresh water flow of 130 L/day. Levels of nitrate (NO_3_^–^) were monitored to avoid stress. Each replicate experiment included four treatments in separate tanks as follows: (1) susceptible shedders infecting susceptible cohabitants (S => S), (2) susceptible infecting resistant (S => R), (3) resistant infecting susceptible (R => S) and (4) resistant shedders infecting resistance cohabitants (R => R). Each tank was populated at day 1 with a combination of 20–22 virus injected shedders and 38–44 naïve cohabitants according to the four treatment combinations. Naïve shedder fish were injected intra-peritoneally (IP) with 0.2 mL containing 36,700 TCID50 of wild-type CyHV-3 virus in PBS solution at day 1 of each replicate. Overall, four replicate experiments were done, each containing these four treatment combinations. Shedders and cohabitants were marked by different caudal fin clipping to allow identification for tissue sampling and mortality recording during an experiment and for counting survivors at the end. Experiments lasted up to 29 days (allowing mortalities to end), during which mortalities, water temperature and water quality were monitored twice a day.

### Tissue samples and DNA extraction

For quantification of viral DNA levels in shedder and cohabitant tissues throughout infection, from each treatment (tank), two live shedders were randomly sampled at day 5, and three live cohabitants were randomly sampled at days 7, 10, 15 and 25. In total, 224 fish were sampled, euthanized by immersion in a high dose of 2-phenoxyethanol, placed on ice and dissected for spleen samples. Samples were kept in 100% ethanol and stored at − 20 °C.

DNA was extracted from spleen using the standard phenol–chloroform extraction protocol. Briefly, about 40 mg of tissue was dried from ethanol, mixed with 600 µL of extraction buffer [50 mM Tris–HCl, pH 8.0, 0.1 M ethylene diamine tetra acetic acid (EDTA), 0.5% sodium dodecyl sulfate (SDS)], and 3 µL Proteinase K (20 mg/mL), and placed for two-hours incubation at 55 °C. After incubation, equal volume of Phenol: Chloroform: Isoamyl-alcohol (25:24:1) was added, mixed and centrifuged for phase separation (13,000 rpm, 10 min). The supernatant phase was taken for one-hour incubation at 37 °C with 10 µL of RNAse A, before repeating the phase separation step. Next, DNA from the supernatant phase was precipitated by adding 2 M NaAcetate (NaAc) to a concentration of 0.2 M, and 2.5 volumes of 100% ethanol, followed by centrifugation (13,000 rpm, 5 min). Liquid was discarded, the DNA pellet was re-dissolved in 400 µL of double-distilled water, and the DNA precipitation process was repeated. Finally, the pellet was washed twice using 500 µL 70% cold ethanol (− 20 °C) and then dissolved in 100 µL of double-distilled sterile water. DNA concentration and quality (optical density, OD260/OD280 ratio) were measured using NanoDrop ND-1000 (Thermo Fisher Scientific) and visually examined by 1.5% Tris/Borate/EDTA (TBE) agarose gel electrophoresis. DNA samples were stored at − 20 °C until used for further analysis.

### Water samples and DNA extraction

For quantification of DNA in tank water, a filtration protocol was adapted from Schroeder et al.^91^ and Sheyn et al.^92^. The volume of water filtered to obtain reliable viral DNA yields was determined by testing various volumes from earlier unrelated CyHV-3 challenge trials and 2 L was found suitable. Water was sampled from each treatment tank at days 5, 7 and 10 of experimental replicates 2, 3 and 4. Water samples were kept at 4 °C until same or next day filtration, through a reusable bottle top filter with a replaceable Isopore™ 0.2 µm filtration membrane (Merck Millipore Ltd.). Membranes containing the filtrate were cut into quarters, and frozen in − 80 °C until DNA extraction.

For DNA extraction, 800 μL of GTE buffer (50 mM glucose, 25 mM Tris–HCl, pH 8.0, 10 mM EDTA) was added to each quarter of the membrane. Further, 100 μL of 0.5 M EDTA and 4 μL of proteinase K solution (20 µg/mL) were added, followed by a two-hour incubation at 65 °C. After adding 200 µL of 10% SDS, the lysate was vortexed to homogenization. Next, equal volume of Phenol: Chloroform: Isoamyl-alcohol (25:24:1) was added. Samples were centrifuged (13,000 rpm, 10 min), and the supernatant was aspirated, split into two 1.5 mL Eppendorf tubes containing about 400 μL each, hence eight sub-samples per one original membrane. Each sub-sample was mixed with NaAc to a final concentration of 0.2 M and 1 mL of 100% ethanol and then centrifuged to precipitate the DNA (13,000 rpm, 5 min). Each resulting DNA pellet was dissolved in 50 µL of double distilled water by 20 min incubation in 55 °C, and the eight sub-samples of each original filter were unified back into a single sample. The joint sample underwent another cycle of precipitation with NaAc and ethanol, washed with 500 µL of freezer-cold 70% ethanol, dried and dissolved in 50 µL of double-distilled water. DNA samples were stored at − 20 °C until used for further analysis.

### Qualitative detection of virus DNA

PCR primers for the viral gene thymidine kinase (TK)^93^ (TK-F: 5′-GGGTTACCTGTACGAG-3′ and TK-R: 5′-CACCCAGTAGATTATGC-3′) were used for primary confirmation of viral presence in DNA samples from tissues and water. As a positive PCR control, primers designed for the fish mitochondrial gene cytochrome oxidase I (COX I)^94^ (cox1-F: 5′-TCAACCACCCACAAAGACATTGGCAC-3′ and cox1-R: 5′-TAGACTTCTGGGTGGCCAAAGAATCA-3′) were used. For PCR amplification, 2 µL of DNA sample (average concentration of 25 ng/µL) were added to a mix containing: 2 µL of GeneAmp™ 10X PCR Gold Buffer (6.6 mM), 2 µL of MgCl2 (25 mM), 1.5 µL dNTPs (6.6 mM), 1 µL of Taq Polymerase, 1 µL primer pair (forward and reverse, 2.5 µM each) and double distilled water to reach a volume of 20 µL. A touchdown PCR profile was used for amplification with the following stages: 94 °C for 3 min, followed by 37 cycles of 94 °C for 30 s, 60–53°C for 1 min (annealing temperature was decreased by 0.5 °C with each of the first 14 cycles, resulting in a final change of − 7 °C), and 72 °C for 1 min, followed by 10 min for final elongation at 72 °C. Positive amplification was examined by electrophoresis of the PCR products on a 1.5% TBE agarose gel stained with ethidium bromide. PCR products were visualized using Gel Doc XR + (BIO-RAD) and using ImageLab software (BIO-RAD). DNAs from CyHV-3 infected fish and naïve fish were used as the positive and negative control, respectively, and a water sample served as a negative PCR control.

When samples yielded no visible PCR product, further nested PCR analysis was performed for detection of low levels of viral DNA. The first stage of amplification used the same protocol as before, with primers used at an annealing temperature of 68–61°C ^95^ (KHV9/5-F: 5′-GACGACGCCGGAGACCTTGTG-3′ and KHV9/5-R: 5′-CACAAGTTCAGTCTGTTCCTCAAC-3′). The PCR products were then diluted × 100 and used as DNA templates for the nested stage with primers used at the same annealing temperature^96^ (KHV-1Fn: 5′-CTCGCCGAGCAGAGGAAGCGC-3′ and KHV-1Rn: 5′-TCATGCTCTCCGAGGCCAGCGG-3′).

### Quantitative detection of virus and fish DNA

Relative CyHV-3 viral load in samples was analyzed by quantitative PCR (qPCR), using novel primers designed to amplify a 165 bp long section of the gene for a membrane protein in ORF 139 (ORF139b) (ORF139b-F: 5′-ATGGTGTCTACCGCCAACTC-3′ and ORF139b-R: 5′-GGTGTTCCTCAACTGGCTGT-3′). Carp DNA was also quantified in the same samples for control and later calibration, using primers designed by^97^ for the elongation factor 1-alpha (EF1-α) gene (EF1-α-F: 5′-CAAGGTCACGAAGTCTGCAC-3′ and EF1-α-R: 5′-CACGAGGTTGGGAAGAACAT-3′). Calibration curves were made for each primer pair to test amplification and the desired DNA template concentration to be used in unknown samples. A virus-positive fish sample was used for both calibration curves, consisting of ten serial dilutions of 4 × each, with an initial concentration of 1250 ng/µL. Primer pairs ORF139b and EF1-α yielded 2.02 and 2.07 amplification efficiency respectively, as well as R^2^ = 1.00 and R^2^ = 0.99, respectively.

For qPCR amplification, a mix was prepared containing 4 µL of water, 2 µL of primer mix (forward and reverse, 2 µM each) and 10 µL Platinum SYBR Green qPCR SuperMix-UDG (Invitrogen) per sample. Of this mix, 16 µL were added to each 96-well plate well and supplemented with 4 µL of DNA (50 ng/µL). The plate was centrifuged and placed in the LightCycler® 96 (Roche). For amplification, a PCR profile was applied, including preincubation at 95 °C for 500 s, followed by an amplification segment (95 °C for 15 s, 63 °C for 30 s, and 72 °C for 15 s with a single fluorescence measurement), repeated for 45 cycles, and a melting segment (95 °C for 10 s, 65 °C for 30 s, and ramp-up to 97 °C, with increments of 0.2 °C/s and continuous fluorescence measurement). Relative concentration (Cq values) was measured at a fluorescence threshold of 0.2 with LightCycler® 96 software version 1.1.0.1320 (Roche), and reported Cq values were used as the data for further analysis.

### Mortality records

For each replicate and treatment, initial day of mortalities, duration of mortalities (days between first and last records) and cumulative percentage of mortality (proportion of dead from total) were recorded. Total fish in each tank excluded the number of fish euthanized and sampled alive. In order to focus on CyHV-3 related mortalities, unexpected mortality records were excluded. Early mortalities, prior to days 3 and 7 for shedders and cohabitants, respectively, were removed as they could have been from causes other than the disease. Late mortalities, post days 12 and 16 for shedders and cohabitants, respectively, were removed since they might have been a result of a secondary infection wave by infected cohabitants rather than by original shedders. Furthermore, due to some variation in mortality initiation days among tanks and replicate experiments, mortalities of all tanks were aligned to the same initiation day by subtracting or adding the difference so that mortalities began at days 5 and 8 for shedders and cohabitants, respectively. This alignment of mortality initiation does not affect cumulative or duration values but allows more reliable comparisons of mortality dynamics differences.

Means of mortality measures across replicates were calculated separately for each fish role (shedder or cohabitant) within each of the four treatments. In figures, the original values were used as they are easier to interpret. For downstream statistical analyses, however, proportional cumulative mortality values were arcsine-transformed [Y’ = Arcsine(√Y)]. Tukey–Kramer honestly significant difference (HSD) test was used to compare final cumulative mortality of the four treatments, separately for shedders and cohabitants.

Survival analyses were performed across replicates, separately for each fish role (shedder or cohabitant). Mortalities post day 12 and 16 for shedders and cohabitants respectively were right-censored. Log-Rank and Wilcoxon tests were used to calculate Chi-square between types of fish (resistant or susceptible) in shedders, and between the four treatments in cohabitants. All statistical analyses were done using the JMP16 software (SAS Institute, Cary, NC, USA).

### Relative DNA load analysis

Relative viral loads were measured in two sample types, spleen tissue of live fish (n = 223) and water filtrate (n = 36), hence, samples occupied several 96-well plates. To account for plate-to-plate and replicate runs variation, the same control samples were added to each plate, including DNA samples from two infected fish (positive for fish and virus PCR), two naïve non-infected fish (positive for fish but not for virus PCR) and two water controls (negative for fish and virus PCR). Each control type was averaged for each qPCR run/plate, and values of different plates were normalized by adding to all plate values the difference in common control values between the specific plate and the reference plate. All DNA samples (whether originating from tissue or water) were analyzed by two PCR primer pairs, one specific to the virus (ORF139b) and another specific to the fish (EF1-α), in at least two technical replicates per sample, and averaged for downstream analyses.

### Viral DNA load from spleen samples

DNA extracted from tissue yielded a combination of fish and viral DNAs. Before further analyses, the viral loads in tissue samples were related to two references using the “-ΔΔCp with efficiency correction” calculation method^98^. The viral Cq values were related first to the fish DNA Cq values, and then to the mean level in treatment S => R at day 7. After calculating these ΔΔCq values, the numbers were Log_10_2^-ΔΔCq^ transformed and two separate data sets for shedders and cohabitants were prepared for statistical analysis. Relative loads in shedders were compared between susceptible or resistant by t-test, as they were sampled only at day 5. Relative loads in cohabitants were analyzed by a three-way ANOVA for the effects of: day (7, 10, 15 and 25), shedder type (susceptible or resistant) and cohabitant type (susceptible or resistant). Following the ANOVA, t-test was used to compare treatments by type of shedder or cohabitant, and Tukey–Kramer HSD was used to compare means of “day” by role (shedder or cohabitant) and by fish type (resistant or susceptible). The Log_10_2^-ΔΔCq^ values were also tested by Tukey–Kramer HSD to compare all samples by day (5, 7, 10, 15 and 25) and by type of fish (susceptible or resistant) or role (shedder and cohabitant).

### Viral load values from water samples

For spleen samples (above), where both viral and fish DNA concentrations scaled-up with tissue size used for extraction, viral DNA load was related to fish DNA load to account for tissue size differences among samples. For water samples, fish DNA can vary by condition and not just scale up with water sample volume. Thus, DNA was extracted from 2 L for all samples, and viral DNA loads were not related to fish DNA loads of the same water sample. Instead, Cq values of virus and fish DNA were related only once, each to its own reference sample, viral DNA load Cq values to mean viral level in treatment R => R at day 5 and fish DNA loads to the mean S => S level at day 5. These relative ΔCq, values were Log_10_2^–ΔCq^ transformed and two separate datasets for each DNA type were prepared. Correlations were tested between viral and fish DNA loads in the same samples, between relative viral DNA load and number of mortalities and between relative fish DNA loads and number of mortalities. Number of mortalities for these correlations was calculated as the sum of day before, of, and day after the water sampling day. We chose to sum mortalities over three days around water sampling, as the water exchange rate made a longer duration less relevant. In addition, relative values from both DNA types were analyzed by a three-way ANOVA for the effect of: sampling day, shedder type and cohabitant type. A t-test was used to compare shedder types or cohabitant types by themselves. Tukey–Kramer HSD and multiple pairwise comparisons student’s *t* test were used to compare among means of day, and to compare between resistant and susceptible fish by their role (shedder or cohabitant).

### Supplementary Information

Supplementary Figure 1.

## Data Availability

All data are included in the manuscript and in its supplementary materials on the journal website.

## References

[1] Fumagalli M (2011). Signatures of environmental genetic adaptation pinpoint pathogens as the main selective pressure through human evolution. PLoS Genet..

[2] Harvell D (2004). Ecology and evolution of host-pathogen interactions in nature. Am. Nat..

[3] Hoye BJ, Fenton A (2018). Animal host-microbe interactions. J. Anim. Ecol..

[4] Buchholz R, Jones Dukes MD, Hecht S, Findley AM (2004). Investigating the Turkey’s ‘snood’ as a morphological marker of heritable disease resistance. J. Anim. Breed. Genet..

[5] Gunjal, K. & Senahoun, J. Assessing the impact of infectious disease outbreaks on agriculture and food security: The case of the Ebola virus disease outbreak in West Africa. In *ICAS VII 2016 Seventh Int. Conf. Agric. Stat. Proc.*10.1481/icasVII.2016.g45d (2017).

[6] Junker, F., Ilicic-Komorowska, J. & Van Tongeren, F. *Impact of Animal Disease Outbreaks and Alternative Control Practices on Agricultural Markets and Trade: The Case of FMD*, Vol. 19 10.1787/18156797 (2009).

[7] Paarlberg PL, Lee JG, Seitzinger AH (2005). Economic modeling of livestock disease outbreaks. Int. Food Agribus. Manag. Rev..

[8] Thompson D (2002). Economic costs of the foot and mouth disease outbreak in the United Kingdom in 2001. Rev. Sci. Tech. Int. Off. Epizoot..

[9] Tomley FM, Shirley MW (2009). Livestock infectious diseases and zoonoses. Philos. Trans. R. Soc. B Biol. Sci..

[10] Lafferty KD (2015). Infectious diseases affect marine fisheries and aquaculture economics. Annu. Rev. Mar. Sci..

[11] Walker PJ, Winton JR (2010). Emerging viral diseases of fish and shrimp. Vet. Res..

[12] Adams LG, Templeton JW (1998). Genetic resistance to bacterial diseases of animals. Rev. Sci. Tech. OIE.

[13] Robinson NA (2022). Applying genetic technologies to combat infectious diseases in aquaculture. Rev. Aquac..

[14] Stear M, Nikbakht G, Matthews L, Jonsson N (2012). Breeding for disease resistance in livestock and fish. Anim. Sci. Rev..

[15] Bisset SA, Morris CA (1996). Feasibility and implications of breeding sheep for resilience to nematode challenge. Int. J. Parasitol..

[16] Dove MC, Nell JA, Mcorrie S, O’connor WA (2013). Assessment of Qx and winter mortality disease resistance of mass selected Sydney rock oysters, *Saccostrea*
*glomerata* (Gould, 1850), in the Hawkesbury river and Merimbula lake, NSW Australia. J. Shellfish Res..

[17] Wolc A (2013). Genome-wide association study for Marek’s disease mortality in layer chickens. Avian Dis..

[18] Bartha I (2013). A genome-to-genome analysis of associations between human genetic variation, HIV-1 sequence diversity, and viral control. eLife.

[19] Cherry S, Silverman N (2006). Host-pathogen interactions in drosophila: New tricks from an old friend. Nat. Immunol..

[20] Johansen LH (2011). Disease interaction and pathogens exchange between wild and farmed fish populations with special reference to Norway. Aquaculture.

[21] Murray AG, Peeler EJ (2005). A framework for understanding the potential for emerging diseases in aquaculture. Prev. Vet. Med..

[22] Pulkkinen K (2010). Intensive fish farming and the evolution of pathogen virulence: The case of columnaris disease in Finland. Proc. R. Soc. B Biol. Sci..

[23] Ødegård J, Baranski M, Gjerde B, Gjedrem T (2011). Methodology for genetic evaluation of disease resistance in aquaculture species: Challenges and future prospects. Aquac. Res..

[24] Pevzner IY, Stone HA, Nordskog AW (1981). Immune response and disease resistance in chickens: I. Selection for high and low titer to *Salmonella*
*pullorum* antigen1. Poult. Sci..

[25] Pevzner IY, Kujdych I, Nordskog AW (1981). Immune response and disease resistance in chickens. II. Marek’s disease and immune response to GAT1. Poult. Sci..

[26] Røed KH, Fevolden S-E, Fjalestad KT (2002). Disease resistance and immune characteristics in rainbow trout (*Oncorhynchus*
*mykiss*) selected for lysozyme activity. Aquaculture.

[27] Bishop SC, Woolliams JA (2010). On the genetic interpretation of disease data. PLoS One.

[28] Chevassus B, Dorson M (1990). Genetics of resistance to disease in fishes. Aquaculture.

[29] Fjalestad KT, Gjedrem T, Gjerde B (1993). Genetic improvement of disease resistance in fish: An overview. Aquaculture.

[30] Doeschl-Wilson AB, Bishop SC, Kyriazakis I, Villanueva B (2012). Novel methods for quantifying individual host response to infectious pathogens for genetic analyses. Front. Genet..

[31] Doeschl-Wilson AB, Knap PW, Opriessnig T, More SJ (2021). Review: Livestock disease resilience: From individual to herd level. Animal.

[32] Råberg L, Sim D, Read AF (2007). Disentangling genetic variation for resistance and tolerance to infectious diseases in animals. Science.

[33] Restif O, Koella JC (2004). Concurrent evolution of resistance and tolerance to pathogens. Am. Nat..

[34] Roy BA, Kirchner JW (2000). Evolutionary dynamics of pathogen resistance and tolerance. Evolution.

[35] Albers GAA (1987). The genetics of resistance and resilience to *Haemonchus*
*contortus* infection in young merino sheep. Int. J. Parasitol..

[36] Lipschutz-Powell D, Woolliams JA, Bijma P, Doeschl-Wilson AB (2012). Indirect genetic effects and the spread of infectious disease: Are we capturing the full heritable variation underlying disease prevalence?. PLoS One.

[37] Shchelkunov IS, Schchelkunova TI (1990). Infectivity experiments with *Cyprinus*
*carpio* iridovirus (CCIV), a virus unassociated with carp gill necrosis. J. Fish Dis..

[38] Velthuis AGJ, Stockhofe N, Vermeulen TMM, Kamp EM (2002). Transmission of *Actinobacillus*
*pleuropneumoniae* in pigs is characterized by variation in infectivity. Epidemiol. Infect..

[39] Gopinath S, Lichtman JS, Bouley DM, Elias JE, Monack DM (2014). Role of disease-associated tolerance in infectious superspreaders. Proc. Natl. Acad. Sci. U.S.A..

[40] Lloyd-Smith JO, Schreiber SJ, Kopp PE, Getz WM (2005). Superspreading and the effect of individual variation on disease emergence. Nature.

[41] Medzhitov R, Schneider DS, Soares MP (2012). Disease tolerance as a defense strategy. Science.

[42] Paull SH (2012). From superspreaders to disease hotspots: Linking transmission across hosts and space. Front. Ecol. Environ..

[43] Anche MT, Bijma P, De Jong MCM (2015). Genetic analysis of infectious diseases: Estimating gene effects for susceptibility and infectivity. Genet. Sel. Evol..

[44] Chase-Topping ME (2021). Impact of vaccination and selective breeding on the transmission of infectious salmon anemia virus. Aquaculture.

[45] Lipschutz-Powell D (2012). Bias, accuracy, and impact of indirect genetic effects in infectious diseases. Front. Genet..

[46] Anacleto O (2019). Genetic differences in host infectivity affect disease spread and survival in epidemics. Sci. Rep..

[47] Anacleto O, Garcia-Cortés LA, Lipschutz-Powell D, Woolliams JA, Doeschl-Wilson AB (2015). A novel statistical model to estimate host genetic effects affecting disease transmission. Genetics.

[48] Griffing B (1967). Selection in reference to biological groups. I. Individual and group selection applied to populations of unordered groups. Aust. J. Biol. Sci..

[49] Tsairidou S, Anacleto O, Woolliams JA, Doeschl-Wilson AB (2019). Enhancing genetic disease control by selecting for lower host infectivity and susceptibility. Heredity.

[50] FAO (2021). FAO Yearbook. Fishery and Aquaculture Statistics 2019.

[51] Ababneh M, Hananeh W, Alzghoul M (2020). Mass mortality associated with koi herpesvirus in common carp in Iraq. Heliyon.

[52] Adamek M (2014). Biology and host response to *Cyprinid* herpesvirus 3 infection in common carp. Dev. Comp. Immunol..

[53] Ariav, R., Tinman, S., Paperna, I. & Bejerano, I. Presented at the EAFP 9th International Conference (1998).

[54] Bretzinger A, Fischer-Scherl T, Oumouna M, Hoffmann R, Truyen U (1999). Mass mortalities in koi carp, *Cyprinus*
*carpio*, associated with gill and skin disease. Bull. Eur. Assoc. Fish Pathol..

[55] Ilouze M, Davidovich M, Diamant A, Kotler M, Dishon A (2011). The outbreak of carp disease caused by CyHV-3 as a model for new emerging viral diseases in aquaculture: A review. Ecol. Res..

[56] Rakus K (2013). Cyprinid herpesvirus 3: An interesting virus for applied and fundamental research. Vet. Res..

[57] Davison AJ (2009). The order Herpesvirales. Arch. Virol..

[58] Gilad O (2003). Molecular comparison of isolates of an emerging fish pathogen, koi herpesvirus, and the effect of water temperature on mortality of experimentally infected koi. J. Gen. Virol..

[59] Hedrick RP (2000). A herpesvirus associated with mass mortality of juvenile and adult koi, a strain of common carp. J. Aquat. Anim. Health.

[60] Ilouze M, Dishon A, Kahan T, Kotler M (2006). Cyprinid herpes virus-3 (CyHV-3) bears genes of genetically distant large DNA viruses. FEBS Lett..

[61] Perelberg A (2003). Epidemiological description of a new viral disease afflicting cultured *Cyprinus*
*carpio* in Israel. Isr. J. Aquac. Bamidgeh.

[62] Dixon PF (2009). Comparison of the resistance of selected families of common carp, *Cyprinus*
*carpio* L., to koi herpesvirus: Preliminary study. J. Fish Dis..

[63] Piačková V (2013). Sensitivity of common carp, *Cyprinus*
*carpio* L., strains and crossbreeds reared in the Czech Republic to infection by cyprinid herpesvirus 3 (CyHV-3; KHV). J. Fish Dis..

[64] Shapira Y (2005). Differential resistance to koi herpes virus (KHV)/carp interstitial nephritis and gill necrosis virus (CNGV) among common carp (*Cyprinus*
*carpio* L.) strains and crossbreds. Aquaculture.

[65] Tadmor-Levi R, Asoulin E, Hulata G, David L (2017). Studying the genetics of resistance to CyHV-3 disease using introgression from feral to cultured common carp strains. Front. Genet..

[66] Zak T, Perelberg A, Magen I, Milstein A, Joseph D (2007). Heterosis in the growth rate of Hungarian-Israeli common carp crossbreeds and evaluation of their sensitivity to Koi herpes virus (KHV) disease. Isr. J. Aquac. Bamidgeh.

[67] Ødegård J (2010). Genetic analysis of common carp (*Cyprinus*
*carpio*) strains. II: Resistance to koi herpesvirus and *Aeromonas*
*hydrophila* and their relationship with pond survival. Aquaculture.

[68] Palaiokostas C (2018). Mapping and sequencing of a significant quantitative trait locus affecting resistance to koi herpesvirus in common carp. G3 Genes Genomes Genet..

[69] Kongchum P (2011). Association between IL-10a single nucleotide polymorphisms and resistance to cyprinid herpesvirus-3 infection in common carp (*Cyprinus*
*carpio*). Aquaculture.

[70] Kongchum P, Hallerman EM, Hulata G, David L, Palti Y (2011). Molecular cloning, characterization and expression analysis of TLR9, MyD88 and TRAF6 genes in common carp (*Cyprinus*
*carpio*). Fish Shellfish Immunol..

[71] Tadmor-Levi R, Hulata G, David L (2019). Multiple interacting QTLs affect disease challenge survival in common carp (*Cyprinus*
*carpio*). Heredity.

[72] Tadmor-Levi R (2019). Different transcriptional response between susceptible and resistant common carp (*Cyprinus*
*carpio*) fish hints on the mechanism of CyHV-3 disease resistance. BMC Genom..

[73] Glass EJ (2012). The molecular pathways underlying host resistance and tolerance to pathogens. Front. Genet..

[74] Muir WM, Aggrey SE (2003). Poultry Genetics, Breeding, and Biotechnology.

[75] Netherton CL, Connell S, Benfield CTO, Dixon LK (2019). The genetics of life and death: Virus-host interactions underpinning resistance to African swine fever, a viral hemorrhagic disease. Front. Genet..

[76] Saura M (2019). Disentangling genetic variation for resistance and endurance to scuticociliatosis in turbot using pedigree and genomic information. Front. Genet..

[77] Yáñez JM, Houston RD, Newman S (2014). Genetics and genomics of disease resistance in salmonid species. Front. Genet..

[78] Chase-Topping M, Gally D, Low C, Matthews L, Woolhouse M (2008). Super-shedding and the link between human infection and livestock carriage of Escherichia coli O157. Nat. Rev. Microbiol..

[79] Wong G (2015). MERS, SARS, and Ebola: The role of super-spreaders in infectious disease. Cell Host Microbe.

[80] Bergmann SM (2010). Goldfish (*Carassius*
*auratus*
*auratus*) is a susceptible species for koi herpesvirus (KHV) but not for KHV disease (KHVD). Bull. Eur. Assoc. Fish Pathol..

[81] Eide KE (2011). Investigation of koi herpesvirus latency in koi. J. Virol..

[82] Reed A (2017). Detection of ORF6 protein associated with latent KHV infection. Virology.

[83] Xu JR (2013). Analysis of koi herpesvirus latency in wild common carp and ornamental koi in Oregon, USA. J. Virol. Methods.

[84] Dishon A, Davidovich M, Ilouze M, Kotler M (2007). Persistence of cyprinid herpesvirus 3 in infected cultured carp cells. J. Virol..

[85] Gaede L (2017). Koi herpesvirus infection in experimentally infected common carp *Cyprinus*
*carpio* (Linnaeus, 1758) and three potential carrier fish species *Carassius*
*carassius* (Linnaeus, 1758); *Rutilus*
*rutilus* (Linnaeus, 1758); and *Tinca*
*tinca* (Linnaeus, 1758) by quantita. J. Appl. Ichthyol..

[86] Perelberg A, Ronen A, Hutoran M, Smith Y, Kotler M (2005). Protection of cultured *Cyprinus*
*carpio* against a lethal viral disease by an attenuated virus vaccine. Vaccine.

[87] Ronen A (2005). Prevention of a mortal disease of carps induced by the carp interstitial and gill necrosis virus (CNGV) in Israel. Bull. Fish Agen. Suppl..

[88] Ronen A (2003). Efficient vaccine against the virus causing a lethal disease in cultured *Cyprinus*
*carpio*. Vaccine.

[89] Doeschl-Wilson AB (2011). Implications of host genetic variation on the risk and prevalence of infectious diseases transmitted through the environment. Genetics.

[90] Springbett AJ, MacKenzie K, Woolliams JA, Bishop SC (2003). The contribution of genetic diversity to the spread of infectious diseases in livestock populations. Genetics.

[91] Schroeder DC, Oke J, Malin G, Wilson WH (2002). Coccolithovirus (Phycodnaviridae): Characterisation of a new large dsDNA algal virus that infects *Emiliana*
*huxleyi*. Arch. Virol..

[92] Sheyn U (2018). Expression profiling of host and virus during a coccolithophore bloom provides insights into the role of viral infection in promoting carbon export. ISME J..

[93] Bercovier H (2005). Cloning of the koi herpesvirus (KHV) gene encoding thymidine kinase and its use for a highly sensitive PCR based diagnosis. BMC Microbiol..

[94] Ward RD, Zemlak TS, Innes BH, Last PR, Hebert PDN (2005). DNA barcoding Australia’s fish species. Philos. Trans. R. Soc. B Biol. Sci..

[95] Gilad O (2002). Initial characteristics of koi herpesvirus and development of a polymerase chain reaction assay to detect the virus in koi, *Cyprinus*
*carpio* koi. Dis. Aquat. Organ..

[96] Bergmann SM, Kempter J, Sadowskim J, Fichtne D (2006). First detection, confirmation and isolation of koi herpesvirus (KHV) in cultured common carp (*Cyprinus*
*carpio* L.) in Poland. Bull. Eur. Assoc. Fish Pathol..

[97] Bar I, Kaddar E, Velan A, David L (2013). Melanocortin receptor 1 and black pigmentation in the Japanese ornamental carp (*Cyprinus*
*carpio* var. Koi). Front. Genet..

[98] Pfaffl MW (2001). A new mathematical model for relative quantification in real-time RT–PCR. Nucleic Acids Res..

